# *NRL*^−/−^ gene edited human embryonic stem cells generate rod‐deficient retinal organoids enriched in S‐cone‐like photoreceptors

**DOI:** 10.1002/stem.3325

**Published:** 2021-01-19

**Authors:** Elisa Cuevas, Daniel L. Holder, Ashwak H. Alshehri, Julie Tréguier, Jörn Lakowski, Jane C. Sowden

**Affiliations:** ^1^ UCL Great Ormond Street Institute of Child Health University College London and NIHR Great Ormond Street Hospital Biomedical Research Centre London UK; ^2^ Centre for Human Development, Stem Cells and Regeneration University of Southampton Southampton UK

**Keywords:** cone photoreceptor, NRL, optic vesicles, retinal organoids, stem cells

## Abstract

Organoid cultures represent a unique tool to investigate the developmental complexity of tissues like the human retina. NRL is a transcription factor required for the specification and homeostasis of mammalian rod photoreceptors. In Nrl‐deficient mice, photoreceptor precursor cells do not differentiate into rods, and instead follow a default photoreceptor specification pathway to generate S‐cone‐like cells. To investigate whether this genetic switch mechanism is conserved in humans, we used CRISPR/Cas9 gene editing to engineer an NRL‐deficient embryonic stem cell (ESC) line (*NRL* ^−/−^), and differentiated it into retinal organoids. Retinal organoids self‐organize and resemble embryonic optic vesicles (OVs) that recapitulate the natural histogenesis of rods and cone photoreceptors. *NRL* ^−/−^ OVs develop comparably to controls, and exhibit a laminated, organized retinal structure with markers of photoreceptor synaptogenesis. Using immunohistochemistry and quantitative polymerase chain reaction (qPCR), we observed that *NRL* ^−/−^ OVs do not express *NRL*, or other rod photoreceptor markers directly or indirectly regulated by NRL. On the contrary, they show an abnormal number of photoreceptors positive for S‐OPSIN, which define a primordial subtype of cone, and overexpress other cone genes indicating a conserved molecular switch in mammals. This study represents the first evidence in a human in vitro ESC‐derived organoid system that *NRL* is required to define rod identity, and that in its absence S‐cone‐like cells develop as the default photoreceptor cell type. It shows how gene edited retinal organoids provide a useful system to investigate human photoreceptor specification, relevant for efforts to generate cells for transplantation in retinal degenerative diseases.

## Graphical abstract

Precursor cells differentiate as rod photoreceptor cells upon expression of the NRL gene; in its absence, the default photoreceptor cell type is a blue opsin‐expressing cone. Parental embryonic stem cells differentiated into retinal organoids generate mostly rods and a small proportion of blue and red/green cones, whereas the engineered NRL‐deficient cells generate rod‐less organoids that contain red/green cones and increased numbers of blue cones.

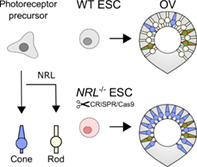

**Significance statement**

Photoreceptor cells located in the retina are essential for vision and their degeneration leads to a large proportion of global blindness. Rod photoreceptors needed for night vision are prevalent, whereas cone photoreceptors needed for high acuity daylight vision are rare. This study has engineered a human pluripotent embryonic stem cell line that lacks NRL gene function. When differentiated in vitro into 3D retinal organoids, the engineered organoids contain cone cells, but no rods. This study showed that NRL is required to differentiate rod photoreceptors and represents a powerful tool to generate enriched populations of cone photoreceptor cells in the laboratory.

## 1 INTRODUCTION

During retinal histogenesis, the different cell types of the neural retina, including the photoreceptor cells, are each born within defined developmental time periods from multipotential retinal progenitor cells.^1^ Rod photoreceptors are generated later and in greater numbers, than the cone photoreceptors needed for daylight vision. The *NRL* gene encodes the neural retina leucine zipper protein,^2^ a conserved bZIP transcription factor that in mouse is initially expressed in nascent rod photoreceptor precursor cells after the terminal progenitor division, and persists in rod photoreceptors thereafter.^3^ Genetic studies have shown that *Nrl* is required for the acquisition of rod identity, differentiation and homeostasis, acting via upregulation of downstream rod target genes, such as the Rhodopsin gene, *Rho*, or *Nr2e3*, which plays a complementary role in the repression of cone specification genes.^4^, ^5^, ^6^, ^7^, ^8^ Nrl acts synergistically with the cone rod homeobox transcription factor, Crx to regulate *Rho* transcription^7^ whereas ectopic expression of *Nrl* under the control of the *Crx* promoter leads to a rod‐only retina.^9^ From an evolutionary perspective, the emergence of the *Nrl* gene is thought to have facilitated the evolution of mammalian rod photoreceptors from short wavelength‐sensitive, S‐cone photoreceptors (also known as blue cones), which are considered to represent an ancient photoreceptor fate.^10^ Conversely, in the retina of *Nrl* ^−/−^ mice, rods are absent and photoreceptors appear cone‐like; *Nrl* ^−/−^ animals exhibit super‐normal cone function mediated by S‐cones, with atypically elevated patterns of blue light detection, concomitant with deficient scotopic vision caused by rod absence.^7^, ^11^, ^12^, ^13^, ^14^ Expression of several key molecular markers are altered in *Nrl* ^−/−^ retinae, which supports the hypothesized model that progenitors generate S‐cones by a default pathway, and that *Nrl* acts like a master regulator required to induce the rod differentiation pathway and suppress the cone fate.^6^, ^10^

In humans, heterozygous missense mutations in the *NRL* gene are associated with dominant retinitis pigmentosa phenotypes^15^, ^16^, ^17^, ^18^, ^19^; gain‐of‐function mutations (at codons 49, 50, 51, 56 in the NRL transactivation domain) lead to reduced NRL phosphorylation and enhanced activation of the rhodopsin promoter.^20^ Such patients present with scotopic vision deficits at a young ages, progressing to loss of photopic response with age; clinical signs that point towards a pattern of early loss of rod photoreceptors, followed by subsequent cone cell death later in life.^19^ By contrast, homozygous and compound heterozygous loss‐of‐function *NRL* mutations cause recessive enhanced S‐cone syndrome (ESCS). ESCS is characterized by an abnormally increased perception of blue light stimuli, coincident with night blindness and abnormal pigmentation patterns.^21^, ^22^ The recessive *NRL* mutations reported in ESCS patients are often nonsense or frameshift mutations.^23^, ^24^, ^25^, ^26^ Unaffected relatives carrying such mutations indicate that heterozygous *NRL* loss‐of‐function is not pathogenic.^24^, ^25^, ^26^ ESCS has also been frequently associated with *NR2E3* mutation. Histological analysis performed on a postmortem retina from a 77‐year‐old individual with ESCS, caused by recessive *NR2E3* mutation (previously diagnosed as retinitis pigmentosa) showed degeneration, lack of rods, and increased numbers of S‐opsin positive cells and reduced L/M‐opsin positive cells.^27^ Similar analysis of NRL‐deficient human retina has not been performed.

Because of its apparent importance in rod generation, models to study NRL function in human development are needed. Retinal organoids generated from human pluripotent stem cells represent an invaluable tool to model human retinal development and pathologies, as they are able to recapitulate typical features of retinogenesis such as an organized multilayered tissue structure, and long human developmental differentiation times.^28^, ^29^, ^30^, ^31^, ^32^ In the present study, we generated a homozygous human embryonic stem cell (ESC) line deficient for *NRL*, taking advantage of CRISPR/Cas9 gene editing to design and introduce a biallelic nonsense mutation into the *NRL* gene. To address whether *NRL* has a conserved role in the establishment of the human rod photoreceptor fate, and if the S‐cone pathway represents a default photoreceptor differentiation pathway as it is in mouse, *NRL* ^−/−^ ESCs were differentiated to form retinal organoids. We analyzed cell identity at different time points, using immunohistochemistry and gene expression via quantitative PCR. We find that *NRL* ^−/−^ optic vesicles (OVs) exhibit a drastic reduction in expression at the RNA and protein level, not only of *NRL*, but also of known NRL‐target genes and characteristic rod markers, particularly at late differentiation time points. Additionally, cone markers are highly upregulated, particularly S‐OPSIN, defining an increased number of S‐cone‐like cells. Our results suggest that the rod photoreceptor identity‐defining function of NRL is conserved in this human in vitro model system, and that in the absence of NRL, human photoreceptor precursors are directed toward a default S‐cone‐like cell fate.

## 2 RESULTS

### 2.1 Generation of *NRL* ^−/−^ ESCs and retinal organoid in vitro differentiation

**FIGURE 1:**
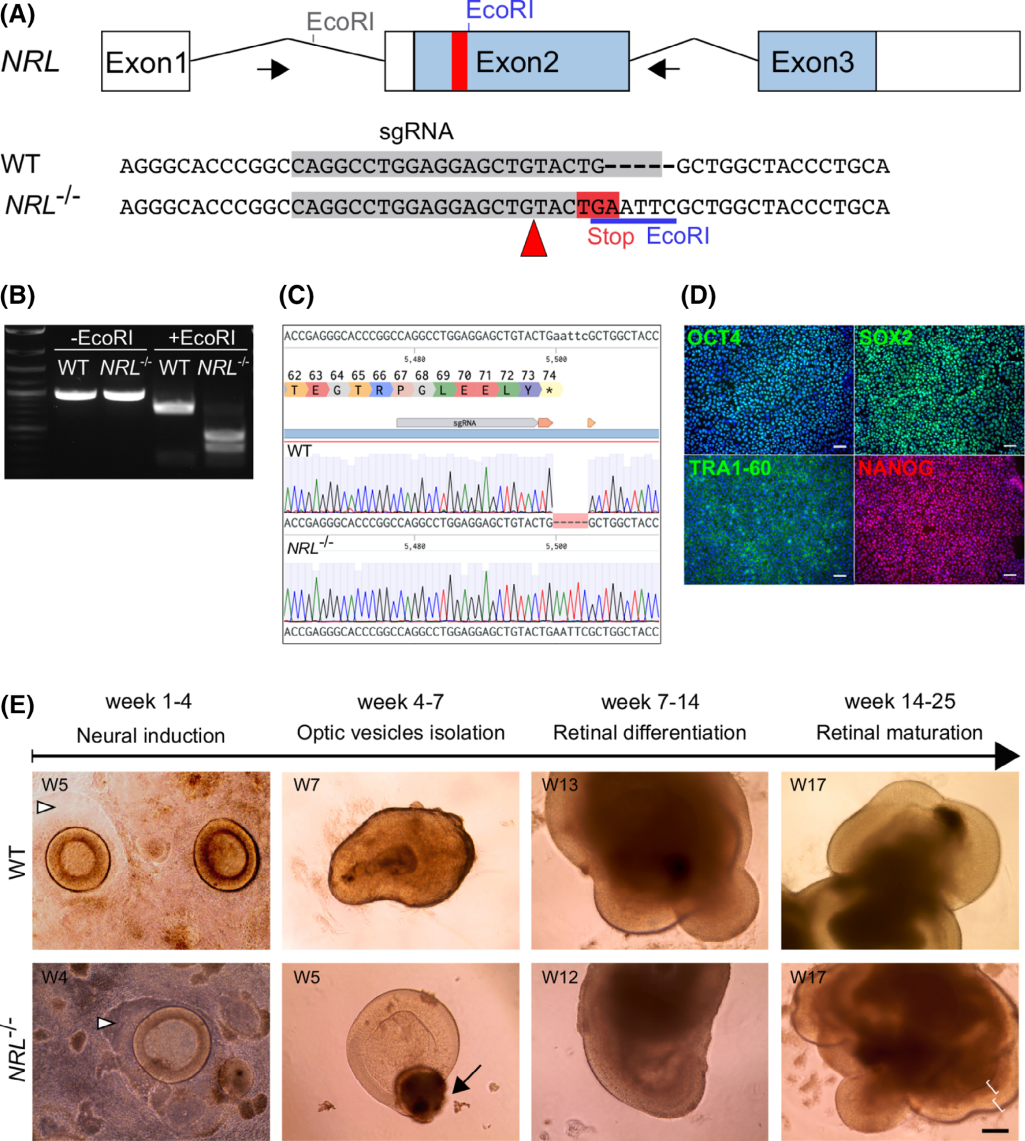
A, The human *NRL* gene comprises three exons, with the translation initiation codon located in the second exon (blue bars). Schematic shows the gene editing strategy using a 127 nucleotide single strand oligonucleotide donor to introduce a nonsense mutation in exon 2 at amino acid position 74 and a new EcoRI restriction site (in blue), 221 bp downstream of the translational start site; a specific guide RNA (gray bar) directed a simultaneous, Cas9‐double strand break. B, Targeted ESCs clones were analyzed by gDNA extraction and PCR‐amplification of the targeted area (indicated by arrows in A), followed by EcoRI digestion to identify donor integration. C, Sanger sequencing confirmed the correct *NRL* gene sequence editing. D, CRISPR‐edited *NRL* ^−/−^ ESCs maintained a healthy morphology and growth rate, and expressed pluripotency markers OCT4, SOX2, TRA‐1‐60 and NANOG, similar to the parental embryonic stem cell line (not shown). DAPI, blue. Scale bar = 100 μm. E, Parental isogenic (wild type [WT]) and *NRL* ^−/−^ ESCs were directed toward retinal differentiation in vitro. Week 1 to 4: Optic vesicles (OVs) displayed a round 3D neuroepithelial structure over a patch of immature retinal pigmented epithelium (arrowheads). Week 4 to 7 OVs were excised and transferred to 96 well plates, to allow maturation as floating organoids. Week 7 to 14: OVs grew in size over time. Week 14 to 25: Lamination was evident from week 15 onward. Scale bar = 200 μm. ESC, embryonic stem cell. DAPI, 4',6‐diamidino‐2‐phenylindole; gDNA, genomic DNA; PCR, polymerase chain reaction

To generate a pluripotent ESC line deficient in NRL, we took advantage of CRISPR/Cas9 technology to target exon number 2, the first coding exon of the *NRL* gene. We designed a small 127 bp single‐stranded donor oligonucleotide (ssODN)^33^ to introduce a stop codon at a.a. position 74, as well as an *EcoRI* restriction site to facilitate screening (Figure 1A). This mutation, c.221_222insAATTC p.(Trp74*) is predicted to yield a truncated version of the NRL N‐terminus, containing only 73 a.a. of the minimal 96 a.a. transactivation domain, while the functional basic Leucine‐zipper DNA binding domain is eliminated. Human ESCs (MShef10; University of Sheffield^34^) were electroporated with a plasmid containing spCas9, a puromycin resistance gene and a single‐guide RNA (sgRNA) scaffold sequence^35^, together with the ssODN; cells were selected and recovered colonies screened using PCR amplification of the targeted region followed by *EcoRI* digestion, to identify the de novo restriction site (Figure 1B). We identified one clone homozygous for the vector insertion, and confirmed its genotype by subcloning and Sanger sequencing analysis (Figure 1C). Off target effects were not detected at three predicted off‐target sites for the sgRNA and the edited line retained a normal karyotype (Figure S1). Targeted cells were expanded, and displayed a normal growth rate and morphology and maintained pluripotency marker expression as determined by immunocytochemistry analysis (Figure 1D).

The *NRL* ^−/−^ ESCs and the parental ESC line (referred to as wild type [WT]) were differentiated toward three‐dimensional retinal organoids using a previously published protocol.^28^ Briefly, ESCs were expanded until confluent, then deprived of pluripotency‐supporting factors fibroblast growth factor (FGF) and transforming growth factor beta (TGFB), followed by a period of neural induction to promote forebrain identity. After 3 weeks, round structures of organized neuroepithelium started to be visible, and were termed OVs. OVs were harvested between week 4 and week 7 and cultured individually in nonadherent 96‐well plates, in retinal differentiation medium (RDM) supplemented with taurine and retinoic acid (see Supporting Information Methods). Using this protocol, we previously showed NRL localized in rod precursors by OV culture week 10, which closely mimics human fetal retinal development.^28^, ^38^, ^39^

No obvious differences were detected between the WT and the *NRL* ^−/−^ lines in terms of ability to generate OVs, vesicle number, or size of vesicles (Figure S2). OVs displayed neuroepithelial morphology as early as differentiation week 3, often growing over a patch of presumptive retinal pigmented epithelium (RPE) (Figure 1E, arrowheads). After isolation, the RPE tissue typically formed a small bundle of pigmented cells at the proximal end of the OVs (arrow). The vesicles continued to grow over several weeks, and in some cases developed additional lobes of neuroepithelium; sometimes more than one organized layer was visible under the light microscope (Figure 1E, brackets).

### 2.2 Characterization of early and mid‐stage *NRL* ^−/−^ OVs

**FIGURE 2:**
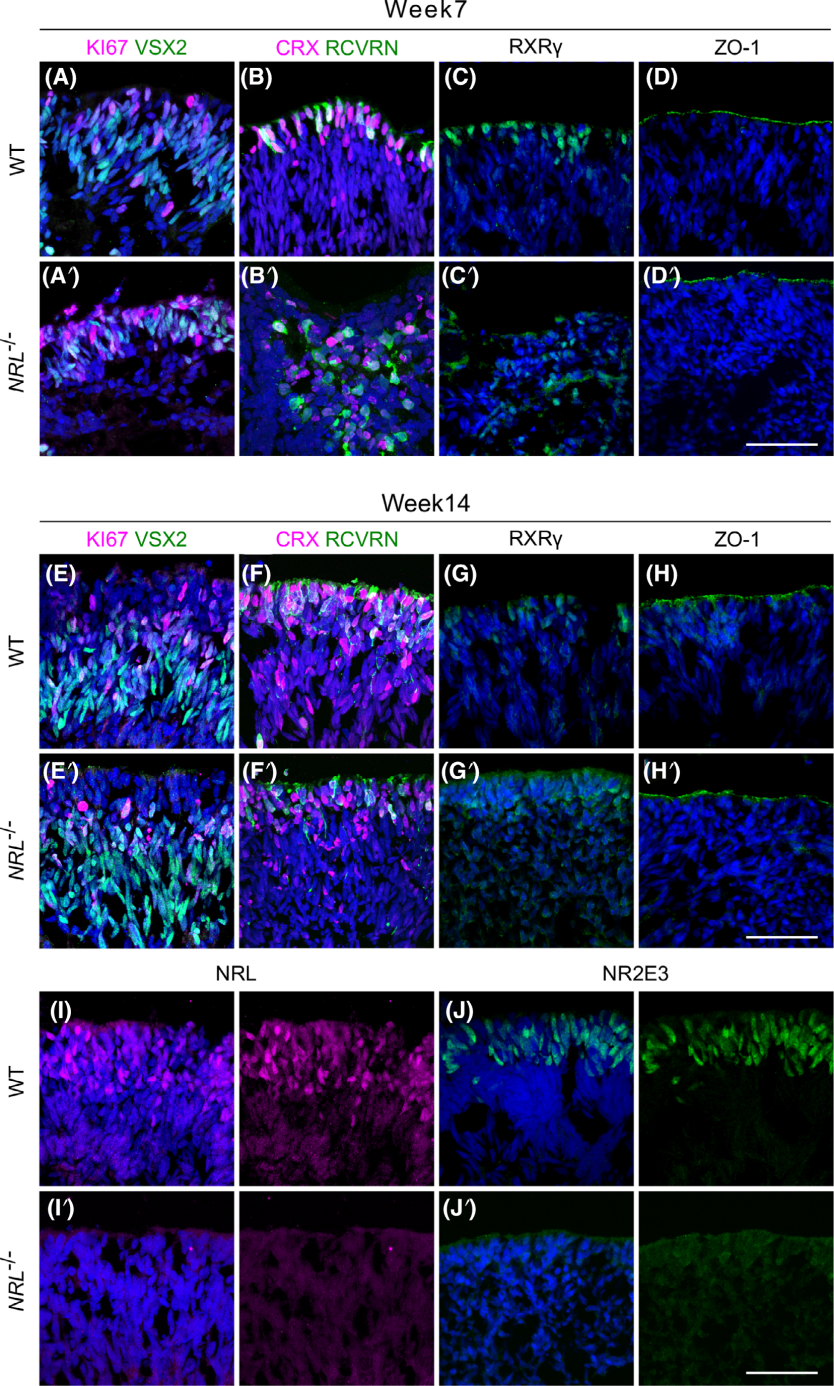
Immunostaining of OV sections using retinal markers showed recapitulation of human retinal development in both WT and *NRL* ^−/−^ OVs. Markers used at week 7 include the retinal progenitor cell marker VSX2, proliferation marker Ki67 (A, A′), early photoreceptor markers CRX and Recoverin (RCVRN) (B, B′), and cone photoreceptor precursor cell marker RXRγ (C, C′). ZO‐1 staining revealed the apical surface and polarization of the OV neuroepithelium (D, D′). At week 14 of differentiation, OVs showed more complex organization with Ki67+ cells, and the localization of VSX2+ cells in a deeper layer, consistent with a transition from retinal progenitors to bipolar cells (E, E′). Pan‐photoreceptor markers CRX and RCVRN stained cells on the outermost layer of the organoids, the presumptive outer nuclear layer (F, F′). RXRγ was used to stain cone‐precursor cells (G, G′) and ZO‐1 to reveal the apical neuroepithelium (H, H′). At week 14 of differentiation, specific markers for rod photoreceptor were used. NRL (I, I′) and NR2E3 (J, J′) are located in the outermost layer corresponding to the photoreceptors in WT control samples, and undetectable in the *NRL* ^−/−^ OVs (N = 2; n = 4). Scale bar = 50 μm. DAPI, blue. DAPI, 4',6‐diamidino‐2‐phenylindole; OV, optic vesicles; WT, wild type

We harvested OVs at week 7 and 14 of differentiation, and performed immunohistological analysis on OV sections to compare the course of differentiation. Both WT and *NRL* ^−/−^ OVs displayed regions of laminated neuroepithelium (WT: n = 96 individual OVs obtained from one independent differentiation passage; *NRL* ^−/−^: n > 400 OVs, from five independent differentiation passages), with an apical outer nuclear layer (ONL)‐like layer. At week 7, the outer layer of cells showed colocalization of the retinal progenitor marker VSX2 and the proliferation maker Ki67 (Figure 2A,A′). The early marker for committed photoreceptor cells CRX was also detectable at this stage, colabeling cells positive for Recoverin (RCVRN), a protein involved in the visual phototransduction cascade (Figure 2B,B′). Some RXRγ‐positive cells were identified, indicating the presence of cone precursors at a comparable stage to that of human fetal development^38^ (Figure 2C,C′). We used ZO‐1 staining to label adherens junctions that revealed apical polarization of the OVs at the presumptive photoreceptor layer (Figure 2D,D′). As expected, no NRL protein was detected at week 7 of differentiation (Figure S2), consistent with OV formation occurring similarly in both WT and NRL^−/−^.

VSX2 is also a marker for bipolar cells that connect with rod photoreceptors and arise later during retinogenesis. Notably, at 14 weeks of differentiation, the Ki67 and VSX2 labeling became more prominent in the layer of cells located immediately underneath the presumptive ONL (Figure 2E,E′). This pattern of expression was consistent with the development of an inner nuclear layer containing bipolar cells. A more organized ONL was apparent by week 14, with CRX and RCVRN staining present in the defined outer layer (Figure 2F,F′). Similarly, RXRγ cone precursor cells continued to organize on the outermost layer of cells, apically labeled with ZO‐1 (Figure 2G,G′,H,H′). Taken together, these immunohistological data support the ability of our gene edited and parental cell lines to form OVs, that display lamination and retinal markers consistent with the human retinogenesis time frame.^36^, ^37^, ^38^

At week 14, NRL was evident in rod precursor cells in the presumptive ONL of the control vesicles, as well as its direct target NR2E3, also expressed in rod photoreceptors and their immature precursors (Figure 2I,J). Strikingly, the *NRL* ^−/−^ retinal organoids showed a downregulation of NRL and NR2E3 immunoreactivity, with a complete absence in the ONL or elsewhere of NRL‐positive cells. Similarly, NR2E3, whose expression is directly regulated by NRL, was not detected in the *NRL* ^−/−^ OVs (Figures 2I′,J′). This indicates the efficiency of the gene editing knockout approach to eliminate functional NRL from retinal three‐dimensional organoids.

### 2.3 Late stage *NRL* ^−/−^ OVs lack rod photoreceptors and generate S‐like cones instead

**FIGURE 3:**
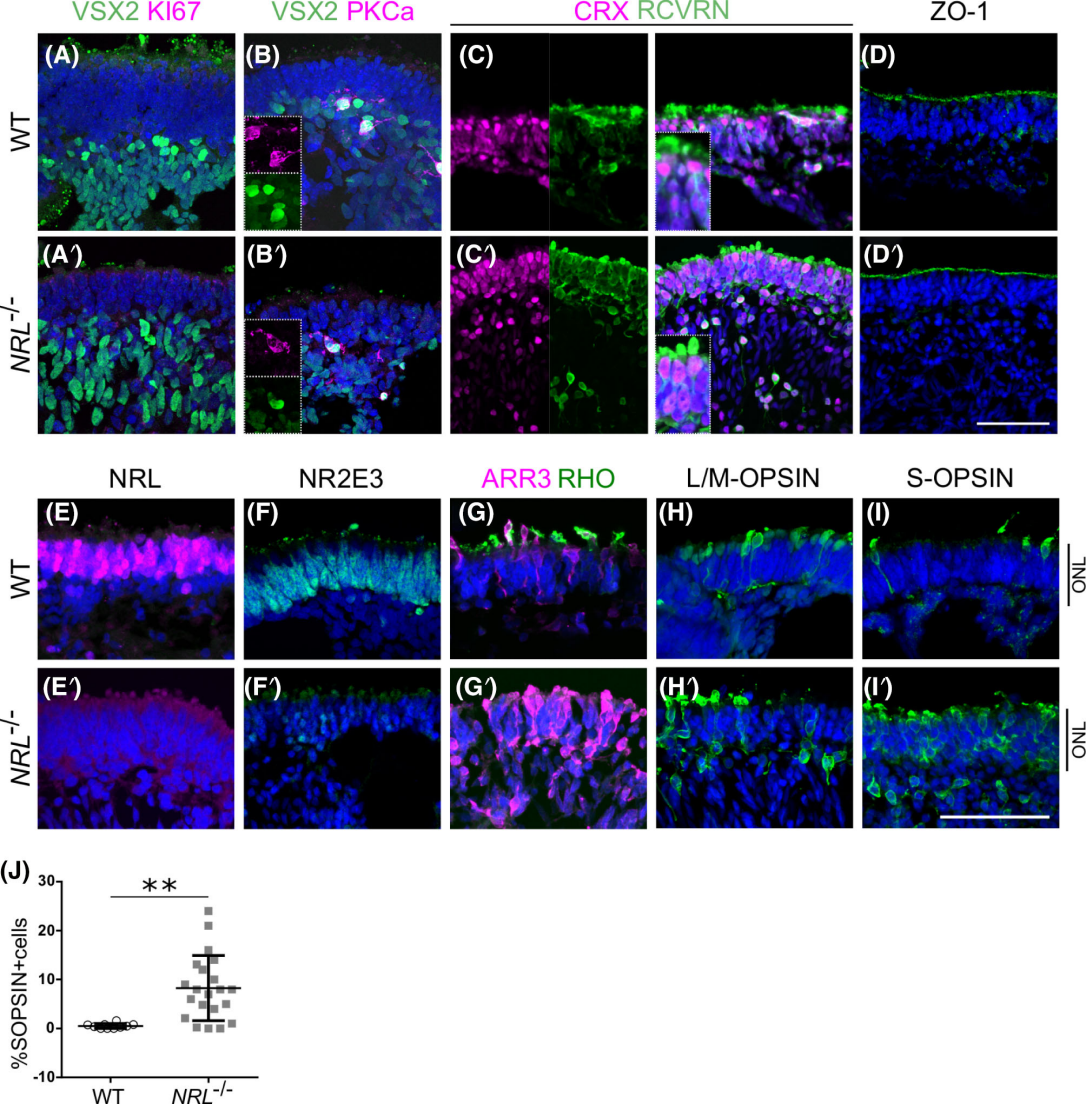
Immunostaining of OV sections at retinal differentiation week 25. OVs present defined lamination and organization of cellular layers, and a differential marker expression between WT and *NRL* ^−/−^ OVs. A,A′, VSX2 was restricted to the cells underlying the ONL, suggesting a presence of bipolar cells that connect with photoreceptors, whereas the proliferation marker Ki67 was undetectable, for both the control and *NRL* ^−/−^ samples. B, B’, VSX2+ population contains a subset of double positive cells for the rod bipolar cell marker PKCa. C, C′, CRX and Recoverin stained the photoreceptor cells populating the ONL of both types of OVs. D, D′, ZO‐1 labeled the apical surface indicating polarization of the OV neuroepithelium. E, NRL localized in the nuclei of the presumptive ONL only in WT OVs. E′, Lack of NRL detected in the *NRL* ^−/−^ line. F, The NRL downstream target NR2E3 localized across the WT OV external cell layer (presumptive ONL) but is absent in the *NRL* ^−/−^ OV (F′). G, Cone photoreceptor marker Cone Arrestin (ARR3) is present in both WT and *NRL* ^−/−^ OVs, which show typical morphology of developing cones cells; but rod‐specific marker Rhodopsin (RHO) was present only in developing photoreceptor cell outer segments in WT OVs and could not be detected in *NRL* ^−/−^ OVs (G′). H, H′, L/M‐OPSIN labels the red and green cone subtypes, and distribution of positive cells is comparable in WT and *NRL* ^−/−^ OVs. I, WT OVs develop a few S‐cones, labeled with S‐OPSIN, in line with human retinal development. I′, Strikingly, *NRL* ^−/−^ OVs present a high proportion of S‐opsin‐positive cells, suggesting a shift in the genetic specification of newly generated photoreceptor cells. Scale bar = 50 μm. DAPI, blue. *NRL* ^−/−^ differentiations, N = 3, n = 4 to 10 samples; WT, N = 2, n = 4. J, Quantification of S‐OPSIN‐ positive cells (mean ± SD, 8.24% ± 1.45 in *NRL* ^−/−^, n = 21 fields from nine samples, three experiments, compared with 0.49% ± 0.15 in WT OVs, n = 10 from three samples, one experiment). ONL, outer nuclear layer; OV, optic vesicles; WT, wild type. DAPI, 4',6‐diamidino‐2‐phenylindole

To characterize the long‐term differentiation of retinal organoids, some of the markers used in early samples were tested in OV cultures at week 25 of differentiation. Very few, Ki67‐positive dividing cells were observed, and VSX2, which labels both retinal progenitor cells and bipolar cells, was at this stage restricted to cells under the ONL, which are presumed to be the latter cell type (Figure 3A,A′). Costaining with ON bipolar cell marker PKCα, confirmed the presence of VSX2+ PKCα+ double positive cells in both WT and *NRL* ^−/−^ OVs (Figure 3B,B′). Virtually all cells within the presumptive ONL expressed the pan‐photoreceptor markers CRX and Recoverin (Figure 3C,C′). OVs showed a distinct organization, with a defined ONL‐like structure of more tightly packed cells at the outermost layer, typically 4 to 6 cells thick. The tight junction marker ZO‐1 remained labeling the apical side of the OVs, indicating the exterior of the laminated structure (Figure 3D,D′).

We analyzed the proportion of VSX2‐positive and Ki67 VSX2 double positive cells within organoids at weeks 14 and 25 (Figure S3A,B). There was no significant difference between the percentage of VSX2‐positive cells between WT and NRL‐deficient OVs at either time point (Figure S3A). The proportion of proliferating Ki67 VSX2‐positive progenitors at 14 weeks (18.50% ± 14.66; 16.44% ± 14.68, mean% ± SE, for WT and NRL^−/−^, respectively) decreased to negligible levels in both lines by week 25 (Figure S3B), suggesting the OVs comprised postmitotic cells by this stage. Variance between samples likely reflected lamination heterogeneity within, and between, organoids.

Because *NRL* is proposed as a crucial player in the establishment and homeostasis of rod photoreceptor identity^7^ we explored how lack of functional protein affected rod and cone photoreceptor differentiation (Figure 3E‐J). As expected, control OVs expressed retinal markers characteristic of rod and cone photoreceptor cell types; cells throughout the ONL showed positive nuclear staining for the rod factors NRL and NR2E3 (Figure 3E,F), indicating the presence of large numbers of rods. Rod‐specific opsin, Rhodopsin (RHO), and cone arrestin (ARR3), expressed in cone photoreceptors, also labeled the cells of the ONL, following a mutually exclusive pattern (Figure 3G). Mature cone markers for cone cell subtypes, L/M‐OPSIN or S‐OPSIN, labeled a proportion of cells in the ONL (Figure 3H,I). The OVs differentiated from *NRL* ^−/−^ ESCs, despite displaying organization with an external ONL formed by closely packed cell bodies, as seen in the controls, showed a drastic change in their protein distribution pattern. Most notable was the disappearance of NRL‐positive cells from the ONL and across the entire OV structure, demonstrating the effective gene knockout strategy (Figure 3E′). Accordingly, NR2E3 labeling disappeared, suggesting loss due to the lack of functional NRL, which is required to activate its expression^7^, ^12^, ^39^ (Figure 3F′). Consistent with the deficiency of rod photoreceptor transcription factors (NRL, NR2E3), the expression of Rhodopsin was undetectable in the *NRL*‐deficient samples, whereas ARR3‐positive cells were present in both WT and *NRL* ^−/−^ samples (Figure 3G,G′).

L/M‐OPSIN labels a subtype of cone cells regulated by pathway components different to that of S‐cones.^40^, ^41^ The immunohistochemical analysis of *NRL* ^−/−^ OVs showed comparable expression of L/M‐OPSIN to the controls (Figure 3H,H′), suggesting that cone genesis and specification of L/M‐opsin cones is not affected by the loss of NRL. Strikingly, the number of S‐OPSIN‐ positive cells in the *NRL* ^−/−^ OVs increased dramatically compared to the control ones, with abundant positive cells found in most areas that present a retinal neuroepithelial ONL structure (Figure 3I′). The quantification of S‐OPSIN‐positive cells at week 25 is shown in Figure 3J. While some OV areas did not develop an ONL and therefore did not have any positive cells, those that generated a laminated retinal structure contained higher numbers of S‐OPSIN‐positive cells in *NRL* ^−/−^ samples compared to those from the WT controls.

### 2.4 Gene expression confirms a tendency to cone gene overexpression at expense of rod markers in *NRL* ^−/−^ OVs

**FIGURE 4:**
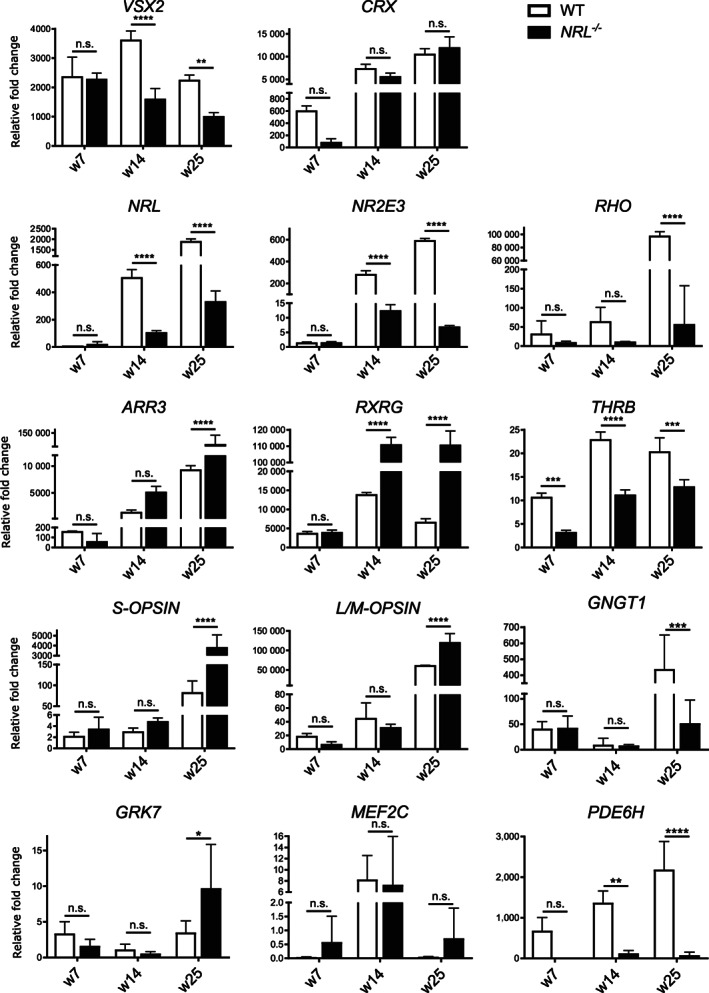
qRT‐PCR analysis of expression of a panel of photoreceptor genes and the neural retinal marker *VSX2* in WT and *NRL* ^−/−^ OVs at three time points, week 7, 14, and 25. Markers used were the pan‐photoreceptor marker cone/rod‐homeobox *CRX*, the rod markers *NRL* and *NR2E3* and cone markers *THRB*, *RXRγ*, *OPN1MW*, *OPN1SW*, and *ARR3* (*NRL* ^−/−^ N = 4 differentiations w7, n = 6; w14, n = 4; w25 n = 6. WT, N = 3 differentiations; n = 4 for each time point). Other genes analyzed were *GNGT1*, expressed in rod outer segments, *GRK7*, involved in the cone photoresponse, cone‐specific phosphodiesterase *PDE6H* and *MEF2C*, putative regulator of rod to cone identity (*NRL* ^−/−^ N = 2 differentiations w7, n = 4; w14, n = 4; w25 n = 4. WT, N = 2 differentiations; n = 4 for each time point). Samples were normalized to GAPDH and d0 (undifferentiated stem cells), plot mean ± SD. **P < .01, ***P < .001, ****P < .0001. GAPDH, glyceraldehyde 3‐phosphate dehydrogenase; OV, optic vesicles; qRT‐PCR, quantitative real time PCR; WT, wild type

To quantify the transcriptional changes caused by the lack of functional NRL, we analyzed the gene expression of a number of retinal genes by quantitative quantitative real time PCR (qRT‐PCR) at weeks 7, 14 and 25 of differentiation (Figure 4). *VSX2* is expressed in retinal progenitor cells at early developmental stages. In our OVs, no differences in *VSX2* expression were observed at week 7. *VSX2* then becomes restricted over time to the bipolar and Müller cell populations in the mature retina.^42^, ^43^, ^44^ Coincident with the temporal acquisition of bipolar cell identity,^45^ *VSX2* expression was significantly reduced in the *NRL* ^−/−^ samples at weeks 14 and 25. The early pan‐photoreceptor marker *CRX* was markedly downregulated at week 7 in the *NRL*‐deficient samples, but not at later time points.

The reduction in *VSX2* gene expression in *NRL* ^−/−^ OVs at later stages was not expected as *VSX2* expression in progenitors precedes *NRL* expression in post mitotic rods, crosstalk between these genes has not been reported, and we showed that the number of VSX2‐positive cells was similar between WT and NRL ^−/−^ OVs at 14 and 25 weeks (Figure S3). As VSX2‐positive cells are postmitotic (Ki67‐negative) at later stages (Figure S3B) together these data suggest that *VSX2* gene expression levels are reduced in differentiating cells (bipolar or Müller glia) in the post mitotic NRL‐deficient retina, while the number of VSX2‐positive cells appears unchanged.

We then studied the expression of a panel of genes involved in rod development and function. *NRL*, as expected, showed a clear downregulation at week 14 in *NRL* ^−/−^ OVs, with expression showing a 10‐fold reduction relative to control by week 25, most likely due to nonsense mediated decay caused by the introduced mutation. The NRL target and rod‐specific transcription factor gene *NR2E3* followed a similar trend, with low, nondifferential expression at week 7, and a drastic reduction in expression in *NRL* ^−/−^ OVs at week 14, falling to a 100‐fold difference in samples by week 25. Finally, this same trend in differential expression between samples was also observed with the rod‐specific photopigment gene *RHO*, whose expression is regulated synergistically by NRL and CRX^5^, ^46^; expression in *NRL* ^−/−^ OVs was reduced relative to controls at week 14, and this difference was highly significant by week 25.

A panel of cone markers was used to investigate whether the increase in the number of cells positive for S‐OPSIN in *NRL* ^−/−^ OVs observed by immunohistochemistry, was coincident with an increase in cone marker expression at the RNA level. By weeks 14 and 25, *ARR3* expression in the edited *NRL* ^−/−^ line was significantly higher than in controls. Similarly, expression of *RXRγ*, which is expressed by cone precursor cells and cooperates with Thyroid Hormone Receptor Beta (THRB) to suppress S‐cone fate and promote M‐cone generation in mouse,^40^ is comparable between mutants and controls at week 7, but significantly upregulated in *NRL* ^−/−^ OVs at later time points. *THRB*, on the contrary, displays an inverted, but weaker pattern, with a reduction in *NRL* ^−/−^ OVs relative to controls at every time point analyzed.

Expression of the cone photopigment genes for *S‐OPSIN* (*OPN1SW*) and *L/M‐OPSIN* (*OPN1MW*), which define the cone subtype populations, were analyzed. In line with the protein observations, *S‐OPSIN* displayed a progressive and marked trend of upregulation upon the loss of *NRL*, significant from week 14 and reaching 100‐fold difference at week 25. *L/M‐OPSIN* showed a significant upregulation at week 25, pointing to a possible influence of NRL loss in the generation of these cone subtypes.

Finally, we examined expression of four genes that were recently reported to be altered in OVs derived from an induced pluripotent stem cell (iPSC) line from a patient with an *NRL* mutation^47^: *GNGT1* (G Protein Subunit Gamma Transducin 1), the gamma subunit of transducin, found in rod outer segments,^48^ was downregulated in the edited *NRL* ^−/−^ line at differentiation week 25; *GRK7*, a retina‐specific G protein‐coupled receptor kinase involved in the cone photoresponse,^49^ was slightly upregulated, at week 25 in the *NRL* ^−/−^ line; *MEF2C*, member of the MADS (MCM1‐agamous‐deficiens serum response factor) family of transcription factors, did not show significant differential expression at any stage; *PDE6H*, a cone‐specific phosphodiesterase, was downregulated in the *NRL* ^−/−^ samples at week 14 and 25.

### 2.5 *NRL*^−/−^OVs establish comparable synaptic connections and show similar glial marker expression at late stages of differentiation compared to WT

**FIGURE 5:**
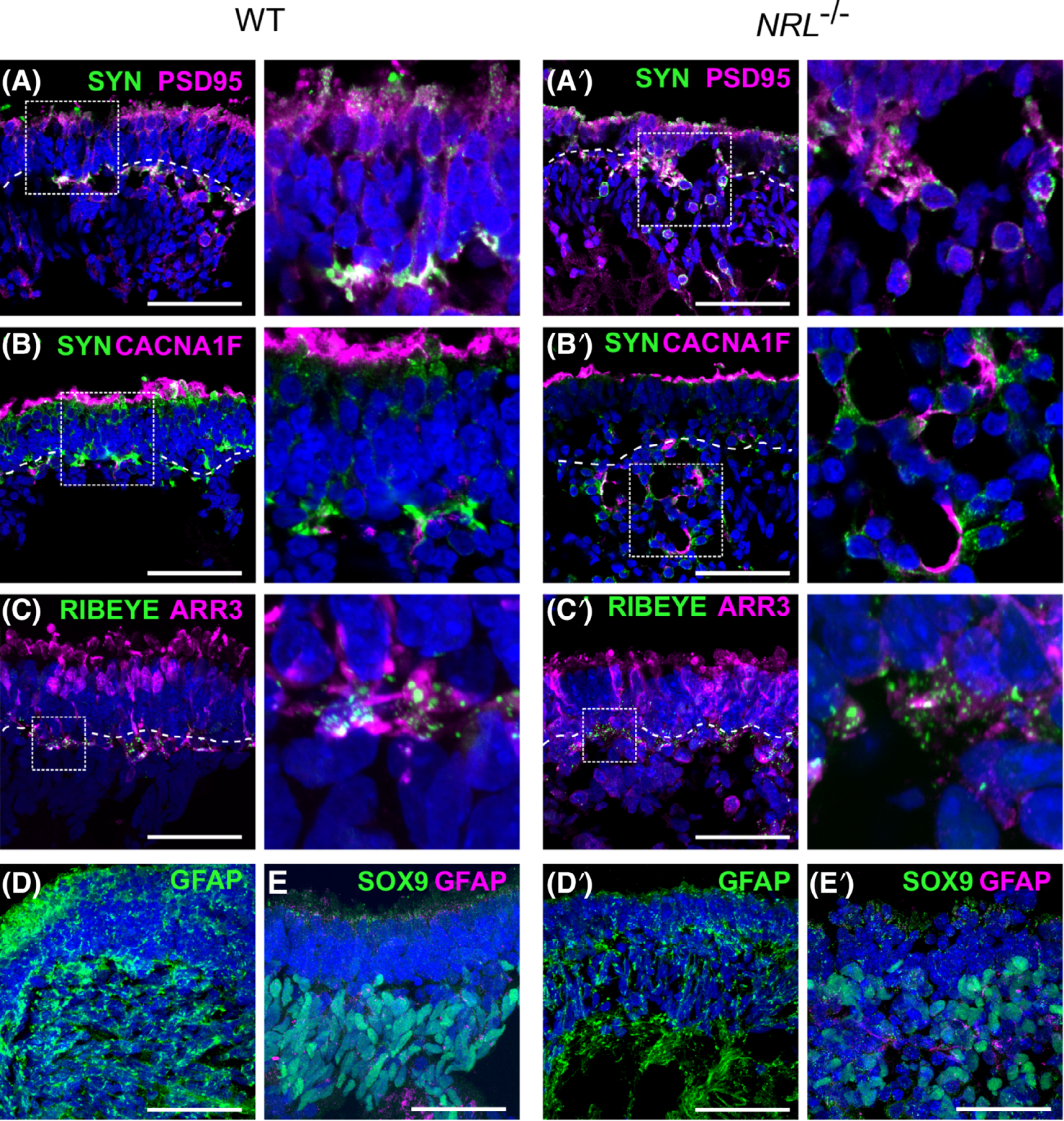
Immunostained OVs at retinal differentiation week 25 from WT and *NRL* ^−/−^ ESCs. A,A′, Synaptic markers Synaptophysin (SYN, green) and PSD95 (magenta) define the putative plexiform layer between presumptive ONL and inner nuclear layer cells. B,B′, Synaptic markers Synaptophysin (green) and calcium channel subunit CACNA1F (magenta) appear adjacent in the processes of the photoreceptor cells. C,C′, Cone marker cone arrestin (ARR3, magenta) and the synaptic protein RIBEYE (green) label photoreceptor layer and underlying synaptic ribbon. Panels to the right are high magnification insets outlined by dotted line. D,D′, Glial marker GFAP (green) with nuclei in blue was used as a marker for stress resulting in gliosis across the OV structure (N = 1, n = 2). E,E′, Müller glia marker SOX9 (green) together with GFAP (magenta) labeling cells of the inner layer. Scale bars = 50 μm. ESC, embryonic stem cell; qRT‐PCR, quantitative real time PCR; ONL, outer nuclear layer; OV, optic vesicles; WT, wild type

Previous studies in the *Nrl* ^−/−^ mouse showed that in the absence of rod photoreceptors, the rod bipolar cells form synaptic connections with cones.^14^ As OVs represent a complex three‐dimensional in vitro retinal culture, capable of developing stratified layers of different cell types^50^ we investigated the ability of the *NRL* ^−/−^ cells to establish interlayer connectivity. We explored the potential for synaptic generation using immunostaining for various synaptic markers on 25‐week‐old OV samples. We detected colocalization of the synaptic vesicle protein Synaptophysin (SYN) and the postsynaptic marker PSD95 basally to the photoreceptor layer, between the ONL and the inner layer of cells, defining a presumptive outer plexiform layer. Both control and NRL‐deficient sections displayed positive staining of these markers in a comparable fashion (Figure 5A,A′). We also used CACNA1F, a calcium channel subunit expressed in photoreceptor cells, and detected positive signal colocalization with Synaptophysin. The pattern of expression for these synaptic markers was indistinguishable between control and *NRL* ^−/−^ sections (Figure 5B,B′). In addition, we used cone arrestin together with RIBEYE, the main component of the synaptic ribbon,^51^ to visualize the cone photoreceptor cell synapses organized in the presumptive outer plexiform layer underlying the polarized photoreceptor cell bodies (Figure 5C,C′).

To investigate whether the lack of NRL might have a detrimental effect on OV integrity, resulting in reactive gliosis, a common feature of retinal dystrophies,^52^ we stained mature retinal organoids sections for activated retinal Müller glia cell marker, glial fibrillary acidic protein (GFAP).^53^ The staining pattern was similar across the two genotypes, being mostly evident beneath the ONL and throughout the core of the organoids. No evidence of enhanced gliosis was observed in the presumptive ONL of NRL‐deficient organoids compared to WT (Figure 5D,D′). To further characterize the inner cell layer of the OVs, we used SOX9, a marker of Müller glia cells, in addition to GFAP.^53^, ^54^ In both WT and *NRL* ^−/−^ samples, the SOX9 positive cells were located internal to the presumptive ONL, consistent with the native retinal organization of Müller glial cells (Figure 5E,E′).

## 3 CONCLUSION

Mammalian retinogenesis is a highly conserved biological process,^55^ although striking differences remain between humans and the mouse models available. The recent expansion of CRISPR/Cas9‐gene editing, human iPSCs and organoid technologies has opened up an extensive platform for researchers to model a myriad of developmental and pathological processes.^56^, ^57^, ^58^, ^59^ Here we report the first *NRL*‐deficient human ESC line, which has been used to investigate the role of NRL in human photoreceptor development and show that human rods share a highly conserved developmental pathway, where NRL is required to form rod photoreceptors, and in its absence increased numbers of photoreceptor precursors acquire an S‐cone‐like phenotype instead. This work allows the future exploration of preclinical potential therapeutic strategies using these supernumerary human cones‐like cells.^60^

## 4 DISCUSSION

By generating a human ESC line with an *NRL* homozygous null mutation, we were able to directly study its effect on retinogenesis in vitro. We introduced a mutation creating a nonsense mutation p.(Trp74*), close to the predicted null *NRL* allele, p.(L75fs), previously reported in ESCS cases with signs of retinal degeneration, clumped pigmentary retinopathy, and preservation of blue cone function.^24^, ^25^ We observed in the organoid model that the loss of functional NRL increases the number of S‐OPSIN positive cells and abolishes NR2E3 expression, which mimics the molecular mechanism behind ESCS; generation of OVs was not affected, consistent with no published reports of disruption of embryonic eye development in ESCS cases. The lack of *NRL* did not appear to affect directly the viability of retinal organoids, since we did not observe an increase in degeneration at early or later culture stages, and *NRL* ^−/−^ OVs have been maintained up to 30 weeks in culture regardless of the cell line genotype (Figure 1 and data not shown). No increase of gliosis was observed in GFAP‐stained OVs, suggesting that the lack of functional NRL in human photoreceptors drives a change of fate in these cells, without compromising the integrity of the retinal organoids (Figure 5D,D′). The OVs mimic neural retinal tissue and include Müller glia cells labeled with SOX9^54^, ^61^ (Figure 5E,E′). ESCS patients often present with clumped pigmentary retinal degeneration, characterized by large clumped pigmented deposits in the RPE of the fundus. Such cases have been described in individuals with recessive mutations in *NR2E3* or *NRL*, suggesting a common genetic basis.^22^, ^24^, ^26^, ^62^, ^63^ The protocol used in our study recapitulates the formation of the neural retina, which lacks a juxtaposed RPE layer, making it not very suitable to address RPE phenotypes like clumped pigmentary retinal degeneration, nevertheless we did not observe any abnormal pigmentation arising within the *NRL* ^−/−^ OV structures.

In mouse, the rodless *Nrl* ^−/−^ retina transiently develops whorls or “rosettes” in the ONL and the photoreceptor outer segments were shorter than those of the WT.^7^, ^11^ These structural changes may result from a conformational alteration due to the loss of rods, leaving supernumerary cones lacking organization and RPE contact, causing degeneration, although in a time‐restricted manner, before stabilization.^11^, ^64^, ^65^ Similar structural abnormalities were described in retinal tissue from a patient affected by *NR2E3* mutations, and attributed to the excessive number of cones produced instead of rods, which pushes the tissue and produces convex folds.^11^, ^27^ In the organoid model, the loss of *NRL* did not cause an abnormal histological organization of the ESC‐derived OVs, nor did it have an effect on epithelial polarity compared to the controls, as seen in the histological analysis using apical marker ZO‐1 (Figure 3C,C′). By week 25, both NRL‐deficient and control organoids lacked mature outer segment formation, but show clear evidence of nascent segment structures extending beyond the ONL (Figure 3G‐I′). Occasionally, OVs presented “rosette‐like” structures, with round doughnut‐like organization of neuroepithelium inside of the organoid; however, we observed this phenomenon in several lines (control, edited, and other PSCs, data not shown) and therefore attributed it to the variability of the OV organization, not the *NRL* mutation.

We report the consequences of edited *NRL* loss‐of‐function mutation in organoids on the expression profile of several retinal genes and markers of cell fate. *VSX2*, expressed by bipolar cells at later time points, is reduced relative to controls. This is noteworthy for several reasons: development of bipolar cells and rod photoreceptors are closely related in mammals^66^; a direct relationship between VSX2 and NRL has not been described, although it has been previously proposed that VSX2 represses photoreceptor differentiation in mouse.^66^, ^67^ Our human data suggest a feedback loop, whereby absence of NRL at later stages leads to reduced *VSX2* gene expression. The cone rod homeobox gene, CRX, that interacts with NRL,^46^ shows comparable levels of expression by week 14 and 25. The dramatically reduced expression of the *NRL* targets *NR2E3* and *RHO* in the NRL‐deficient organoids relative to controls indicating loss of differentiated rod cells is consistent with NRL acting as a key regulator for rod specification. Consequently, cone photoreceptor genes showed an elevated expression in *NRL* ^−/−^ OVs compared to controls. *ARR3*, *RXRƔ*, and *THRB* are expressed earlier in development, and the latter two are known to be involved in cone specification in mouse.^68^ Expression of *ARR3* and *RXRƔ* is higher in NRL‐deficient OVs than controls at the RNA level, which is consistent with an enhanced cone identity of cells in the NRL‐deficient samples. Notably, in contrast to the direct regulation of the *Mef2c* promoter activity by Nrl in mice suggesting its role in rod development,^69^ we found no evidence supporting NRL regulation of *MEF2C*. *MEF2C* expression was unchanged in the human NRL^−/−^ OVs organoids and Kallman et al^47^ showed MEF2C was enriched in cone photoreceptors, suggesting MEF2C may play a different role, and is differently regulated, in humans and mice.

*Nrl* is known to interact with cone‐specific genes to repress their activity, and has been shown to bind the promoter region of *ThrB*, responsible for M‐cone specification^9^; this de‐repression effect might explain the results of reduced *THRB* gene expression in *NRL* ^−/−^ organoid samples. Previous studies in mouse have reported variable impact of *Nrl* loss on expression of M‐cone specific marker *Opn1mw*; earlier studies indicate no changes in Opn1mw protein expression,^7^, ^64^ whereas others report a slight increase in gene expression levels^27^, ^70^ and the presence of both S and M‐OPSIN pigment in the physiological responses of *Nrl* ^−/−^ photoreceptors.^11^, ^13^ Here, we observed a large increase in *OPN1SW* expression and a modest increase in *OPN1MW* gene expression in late time point *NRL* ^−/−^ OVs (Figure 4), although L/M‐OPSIN protein distribution appeared equivalent (Figure 3H,H′). These data suggest a role for *NRL*, whether direct or indirect, in influencing L/M‐cone specification in mammals, in addition to the established repression of S‐cone identity.

Assessment of the ultrastructural, molecular, and electrophysiological features of photoreceptors cells from *Nrl* ^−/−^ mice showed remarkable similarity to *bona‐fide* cones, while other studies suggested the generation of intermediates between rods and cones.^11^, ^13^, ^70^ A study on retinal organoids derived from a patient iPSC line with an *NRL* mutation similar to that engineered here in ESC was recently published.^47^ This study characterized cone and rod photoreceptors using immunostaining and single cell RNA sequencing techniques. Consistent with our work, an increase in S‐OPSIN+ cells was observed in the iPSC line, which lacks expression of rod proteins. Interestingly, the single cell analysis revealed two S‐OPSIN+ populations, one similar to WT S‐cones, and a second one that displays some rod photoreceptor features. Consistent with mouse studies, these results point to the existence of a hybrid population termed “cods” and indicate aspects of human rod photoreceptor specification are independent of NRL.

Most features measured in *Nrl* ^−/−^ cells in the mouse model suggest they are healthy and functional,^11^ and they connect to rod bipolar cells, implying that the type of photoreceptor connections is not pre‐established based on cell type.^14^ We observed that *NRL* ^−/−^ OVs form organized layers that express synaptic markers, similar to control organoids, and therefore it appears that the main consequence of losing functional NRL is the change in photoreceptor identity, without compromising cellular homeostasis. Our system therefore represents an in vitro human model that generates supernumerary S‐cone‐like cells and L/M‐cones, but not rods, rather than a human retinitis pigmentosa model, associated with harmful gain of function mutations and characterized by degenerative features.

Several groups have recently explored tampering with *Nrl* as a therapeutic approach to stimulate the endogenous generation of cones capable of rescuing degeneration and cone‐specific visual function. Using various methods to repress, eliminate or generate a loss‐of‐function *Nrl* mutation in mouse models of retinal degeneration, retinal degeneration was prevented by reprogramming rods to cones, accompanied by a recovery of ONL architecture and an increase in cone marker expression.^71^, ^72^, ^73^, ^74^ Nrl‐deficient cone‐like mouse photoreceptors have also been transplanted subretinally in a model of retinal dystrophy and shown to restore some photopic vision.^75^

The culture of human cells as organoids presents an opportunity to generate virtually unlimited specific cells for transplantation therapy, provided that these are physiologically functional. Photoreceptors must be capable of establishing synapses with second order retinal cells in order to be a relevant product for cell therapy. Previously, a study using *Nrl* ^−/−^ mice reported that expression of the synaptic calcium channel component CACNA1F is altered in the mutant adult, but not newborn retinas, suggesting a role for Nrl in its maturation.^67^ By contrast, Nrl overexpression in mouse results in cone‐deficient retinas that maintain synaptic organization with expression of the postsynaptic protein PSD95.^9^ In the present work, we showed that OVs at 25 weeks, equivalent to a mid‐gestational stage develop distinct layers of cells that organize in a comparable manner to the human retina. The photoreceptor cells in the *NRL* ^−/−^ OVs showed evidence of synaptic connections by organized distribution of several key human synaptic markers (PSD98,^29^, ^32^ synaptophysin,^50^ and RIBEYE^51^ in a presumptive plexiform layer, suggesting the generation of cone cells capable of establishing connectivity across cell layers; Figure 5A,A′,B,B′,C,C′). We did not detect differences in synaptic marker distribution in control and NRL‐deficient OVs suggesting capacity for photoreceptor connectivity was not diminished at this stage in the cone‐rich human organoids, although specific markers for human cone synapses are not available.

In summary, our study indicates the value of using genetically modified cell lines to characterize the retinal developmental events in humans and to generate new disease models for in vitro study. Modified OVs also represent a potential source of abundant cone photoreceptor cells for use in generating a cell product suitable for cone cell replacement transplantation therapy. However, clinical application would be contingent upon regulatory approval of such genetically modified human ESCs.

## 5 METHODS

Additional details are provided in Supporting Information Methods.

### 5.1 ESC culture

Human ESCs were routinely maintained on Laminin‐coated 6 well plates on NutriStem medium. When at 80% to 90% confluence, cells were lifted using EDTA 0.5 mM and replated at 10 000 cells/cm^2^.

### 5.2 CRISPR/Cas9 gene ablation of *NRL*

To generate an NRL‐deficient ESC line, we designed a small asymmetric ssODN^33^ to insert a STOP codon at the a.a. position 74 of *NRL*. We electroporated the ssODN donor together with a plasmid containing the *NRL* sgRNA and a Cas9 sequence in ESCs; selected colonies were screened for the insertion of the donor sequence, and one clone homozygous for the designed mutation (*NRL* ^−/−^) was identified.

### 5.3 Retinal differentiation

Both parental MShef10 and *NRL* ^−/−^ ESCs were subjected to retinal differentiation using a previously published protocol.^28^ Briefly, cells were cultured to confluence and cultured in Neural Inductive Media until beginning to form three‐dimensional OVs. These are picked before week 7 of differentiation and further cultured in RDM.

### 5.4 Immunohistochemistry

OVs were fixed in a 4% paraformaldehyde (PFA) solution, and cryopreserved to generate 12 μm sections. Slides were incubated with the primary antibody O/N at 4°C, followed by secondary antibody incubation 2 hours at R/T and 4',6‐diamidino‐2‐phenylindole (DAPI) counterstaining. Antibodies used are listed in Table S1.

### 5.5 Quantitative PCR

Triplicate samples of independently cultured OVs were isolated at week 7, 14, and 25; undifferentiated ESCs were used as control. RNA was extracted using the RNeasy Micro Kit and cDNA was synthesized using the RevertAid H Minus First Strand cDNA Synthesis Kit. qRT‐PCR was performed using Glyceraldehyde 3‐phosphate dehydrogenase (GAPDH) as reference gene and Ct values were analyzed as previously described.^76^ Primers sequences are in Table S2.

## CONFLICT OF INTEREST

The authors declared no potential conflicts of interest.

## AUTHOR CONTRIBUTIONS

E.C.: conception and design, provision of study material, collection and/or assembly of data, data analysis and interpretation, manuscript writing, final approval of manuscript; J.T.: collection and/or assembly of data; D.H.: conception and design, provision of study material, collection and/or assembly of data, manuscript writing; A.A.: collection and/or assembly of data, data analysis and interpretation; J.L.: conception and design, provision of study material; J.C.S.: conception and design, data analysis and interpretation, manuscript writing, financial support, final approval of manuscript

## Supporting information

**Supplementary Figure 1** CRISPR off‐target effects
**Supplementary Figure 2**. Representative examples of OVs at differentiation week 14 and NRL immunostaining in week 7 OVs
**Supplementary Figure 3**. Proportion of VSX2+ progenitor cells at differentiation week 14 and 25 in WT and NRL^−/−^ OVs
**Table 1**. Antibodies list
**Table 2**. qRT‐PCR primers list
**Table 3**. CRISPR/Cas9 Off‐target effects
**Table 4**. Primers to amplify off‐target regions

## Data Availability

The data that support the findings of this study are available from the corresponding author upon reasonable request.
