# Supplementary material for: NRL−/− gene edited human embryonic stem cells generate rod‐deficient retinal organoids enriched in S‐cone‐like photoreceptors
Source: Stem Cells. 2021 Jan 19;39(4):414–28. doi: 10.1002/stem.3325 (PMC8438615; doi:10.1002/stem.3325)
Supplement: Supplementary file 1 — Supplementary Figure 1 CRISPR off‐target effects Supplementary Figure 2. Representative examples of OVs at differentiation week 14 and NRL immunostaining in week 7 OVs Supplementary Figure 3. Proportion of VSX2+ progenitor cells at differentiation week 14 and 25 in WT and NRL−/− OVs Table 1. Antibodies list Table 2. qRT‐PCR primers list Table 3. CRISPR/Cas9 Off‐target effects Table 4. Primers to amplify off‐target regions [file STEM-39-414-s001.pdf]

A

	Sequence	PAM	Score	Gene	Chr.	Strand	Position	Mismatches	On-target
	CAGGCCTGGAGGAGCTGTAC	TGG	100	ENSG00000129535	chr14	-1	24082633	0	FALSE
1	CAGGTCTGGAGGAGCTGTAG	AAG	5.6592857		chr9	-1	132597790	2	FALSE
2	CAGGCCTGTAGGAGCTGTCC	AGG	1.6621977	ENSG00000171298	chr17	1	80104569	2	FALSE
3	CAGGGCTGGGGGAGCTGTAG	AAG	1.4741582		chr7	-1	43197557	3	FALSE
4	GAACCCTAGAGGAGCTGTAC	CAG	1.4575207		chr1	-1	6462730	4	FALSE

Off-target site #1

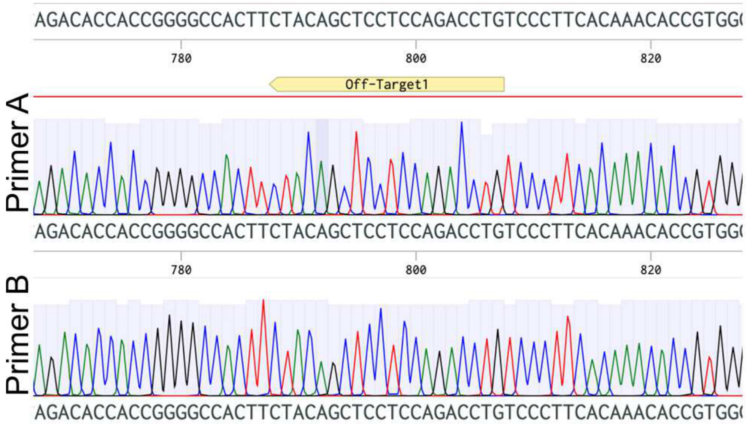

Off-target site #3

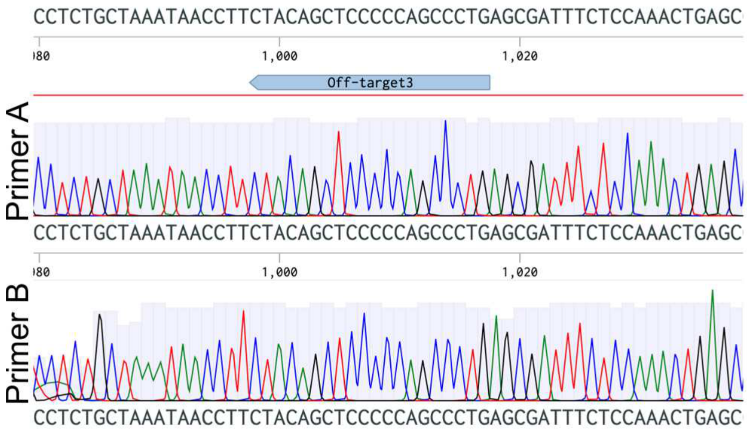

Off-target site #4

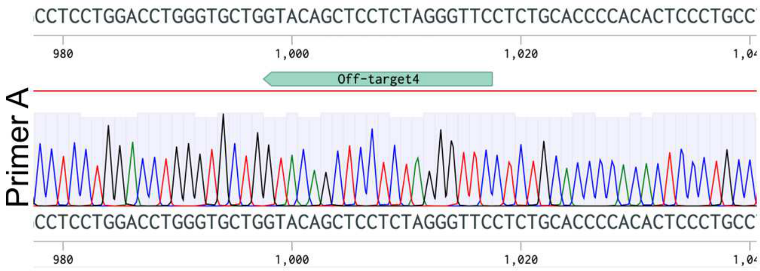

B

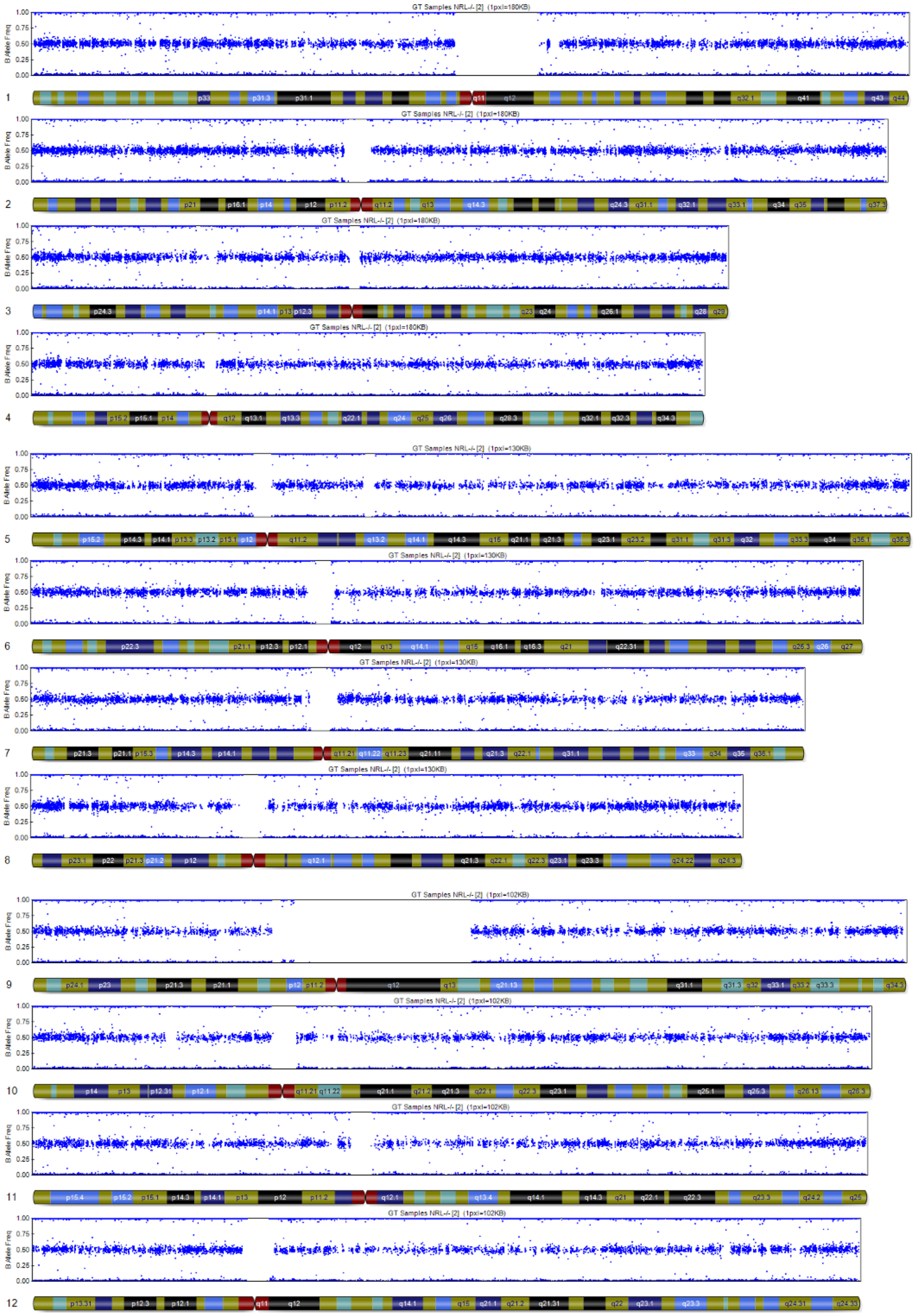
GT Samples NRL-/- [2] (1pxl=180KB)
B Allele Freq
1.00
0.75
0.50
0.25
0.00
1
GT Samples NRL-/- [2] (1pxl=180KB)
2
GT Samples NRL-/- [2] (1pxl=180KB)
3
GT Samples NRL-/- [2] (1pxl=180KB)
4
GT Samples NRL-/- [2] (1pxl=130KB)
5
GT Samples NRL-/- [2] (1pxl=130KB)
6
GT Samples NRL-/- [2] (1pxl=130KB)
7
GT Samples NRL-/- [2] (1pxl=130KB)
8
GT Samples NRL-/- [2] (1pxl=102KB)
9
GT Samples NRL-/- [2] (1pxl=102KB)
10
GT Samples NRL-/- [2] (1pxl=102KB)
11
GT Samples NRL-/- [2] (1pxl=102KB)
12

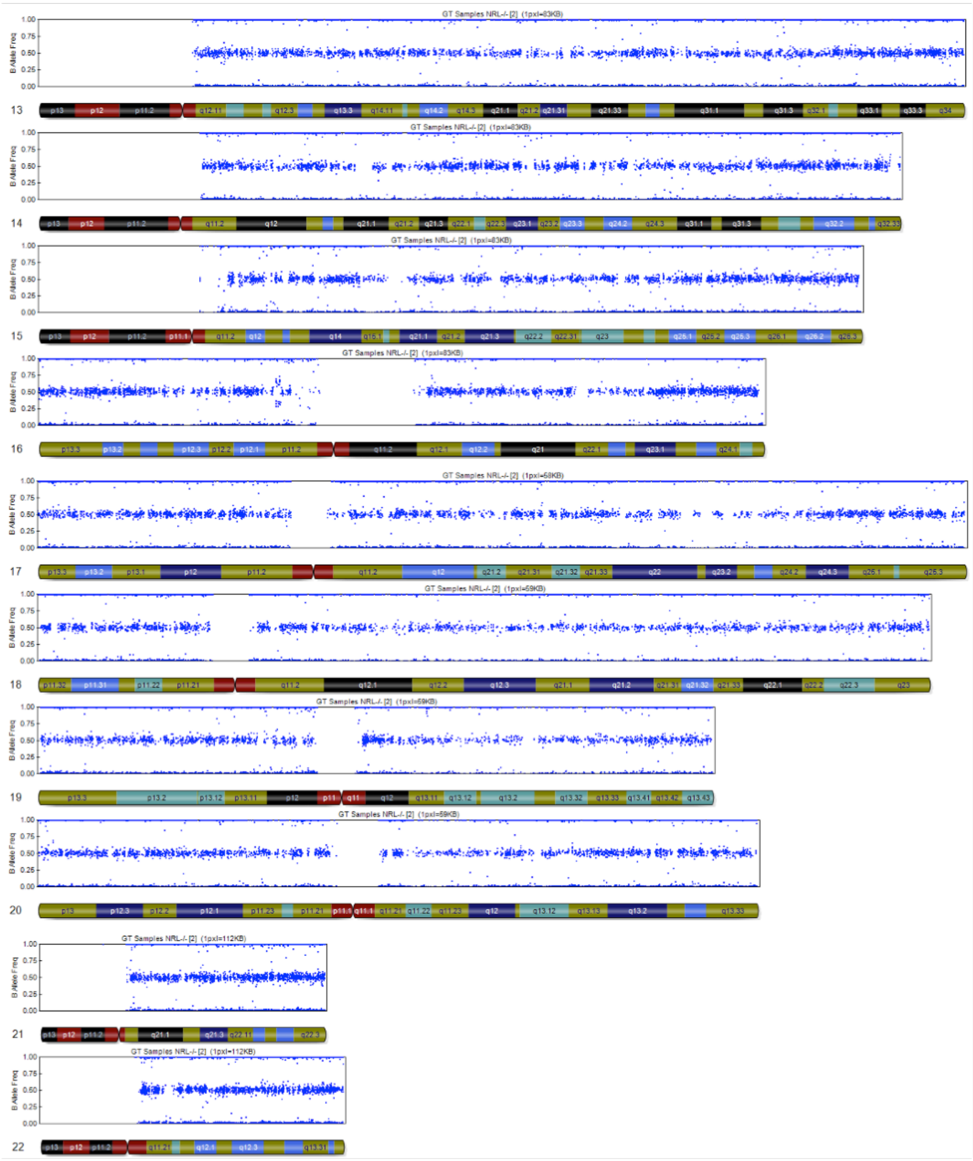

Supplementary Figure 1. CRISPR off-target effects

A. The first four off target sites predicted for *NRL*$^{-/-}$ sgRNA were investigated by amplifying the genomic region via PCR; locations #1, #3 and #4 were amplified and analyzed using Sanger sequencing, with no mismatches observed. Region #2 proved difficult to amplify in edited and non edited genomic DNA.

B. SNP analysis of *NRL*$^{-/-}$ genomic DNA showing a normal karyotype; mapping from Genomestudio 2.0 shows individual SNP markers on the array plotted as a function of the frequency of the minor (B) allele across each chromosome.

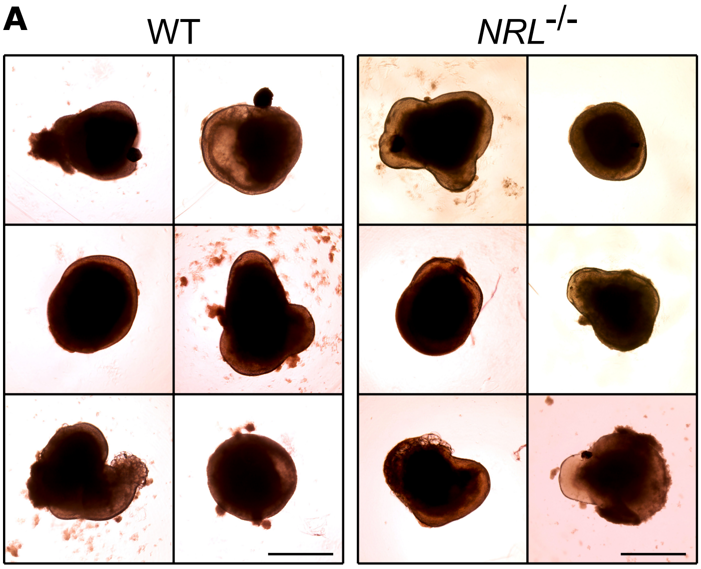

	WT	NRL-/-
N=	8	6
Total OVs	402	505
MEAN	21.81	20.13
SD	21.77	8.05

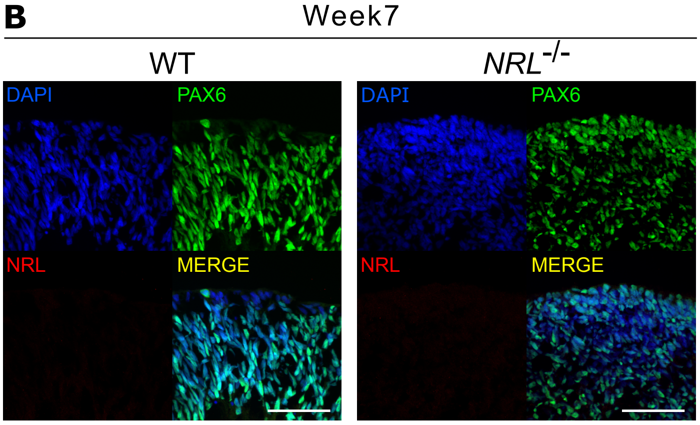

Supplementary Figure 2. Representative examples of OVs at differentiation week 14 and NRL immunostaining in week 7 OVs

A. Bright field images of WT and *NRL*$^{-/-}$ retinal organoids (OVs) at differentiation week 14 in culture, showing variable sizes and morphology; they can be round in shape, or with bulges and lobes, and RPE present. Table summarizes the average number of OVs generated per well (of a 6-well plate) with standard deviation (SD) from independent differentiation experiments (N). No evidence was found for a change in OV generation relative to genotype. **B**. Immunostaining of WT and *NRL*$^{-/-}$ OV sections at differentiation week 7, using early retinal marker PAX6, and NRL. Scale bars; A, 1 mm; B, 50 μm.

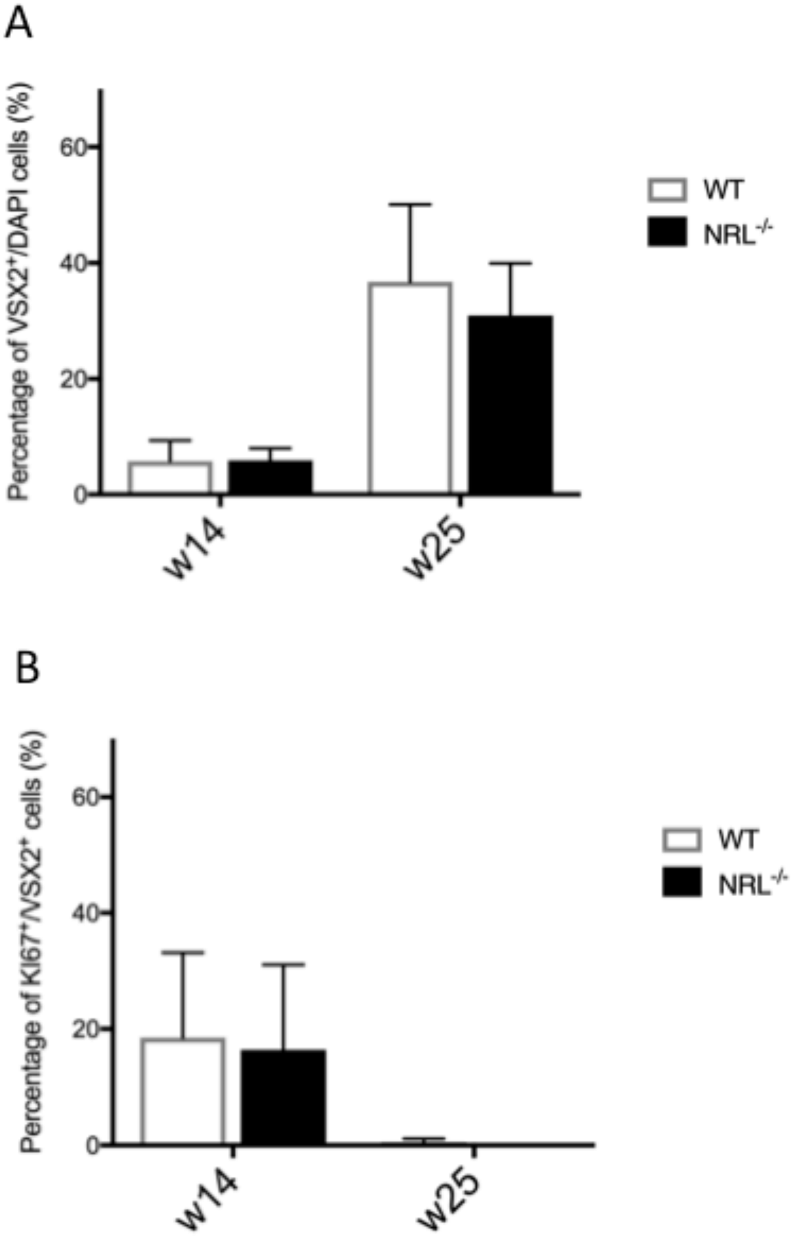

Supplementary Figure 3. Proportion of VSX2+ progenitor cells at differentiation week 14 and 25 in WT and *NRL$^{-/-}$* OVs

A. VSX2+ cells as a percentage of total cell nuclei labelled with DAPI; **B.** Percentage of KI67+ cells as a percentage of VSX2+ cells, in WT and *NRL$^{-/-}$* organoids at week 14 and week 25 (N = 1 differentiation and n = 3 organoids for each genotype). Mean ± SD. No significant difference between WT and and NRL$^{-/-}$ OVs.

Supplementary Methods File

Detailed Methods

ESC culture

The MShef10 embryonic stem cell line (hPSCreg Name, UOSe014-A) generated by the Centre for Stem Cell Biology, University of Sheffield[34] was cultured using standard procedure. Briefly, flasks of 6-well plates were coated with 5 μg/ml Laminin521 (Biolamina, #LN521) in PBS (+Mg/+Ca2+, PBS Life Technologies, #14190-091) at 4°C O/N. Laminin solution was aspirated and the flask covered with pluripotency supportive NutriStem medium (Biological Industries, #05-100-1A). Cells were plated at a density of 10,000 cells/cm^2, fed daily after the first 48 h, and usually passaged every five days, when ~80% confluency was reached. To lift the cells, semi-confluent cultures were washed twice with PBS (-Mg/-Ca^{2+}, PBS Life Technologies #14190-094), and incubated with a 0.5 mM solution of EDTA (Life Technologies, #15575-020) in PBS$^{-/-}$ for 5 minutes at 37° C. Clusters of cells were detached by tapping the flask, resuspended in media, centrifuged for 5 minutes at 200g and counted for replating.

CRISPR/Cas9 gene ablation of *NRL*

To introduce a mutation in the *NRL* gene of MShef10 ESC cells, a small donor was designed to achieve HDR repair following Cas9 double strand break[35]; a phosphorylated 127 bp asymmetric single-stranded donor oligonucleotide (ssODN) (Biolegio) containing a 5 bp sequence change was used to insert a premature STOP codon at a.a. position 74 of *NRL* gene, and a novel EcoRI restriction site was also introduced to facilitate screening for donor integration. The Wellcome Sanger Institute Genome Editing CRISPR finder tool[35] was used to identify the sgRNA sequence (CAGGCCTGGAGGAGCTGTAC). The sgRNA

guide was selected to avoid an alternative translational start site, and with predicted off-target sites having at least two mismatches, reducing the chances of unspecific gene editing events. The guide was cloned under the control of a U6 promoter into a modified version of the pSpCas9(BB)-2A-Puro (PX459) V2.0 (Addgene, # 62988) plasmid, in which the ChB promoter was replaced with an EF1α promoter, in order to optimize expression in human stem cells. 1x 10^6 cells were co-electroporated with 100µmol of ssODN and 10µg Cas9 plasmid using a BTX ECM 830 electroporator (100V, 5ms, 50ms interval, bipolar pulse). 24h after electroporation, cells were subjected to 0.5 µg/ml Puromycin selection for 72h period of puromycin selection (Thermo Fisher, #A1113803). Surviving colonies were collected (36 colonies plus 3 pools of cells), and gDNA extracted from half the sample using QuickExtract (Lucigen, #QE09050). PCR was performed using primers flanking exon 2 and PCR products treated with EcoRI; a double cutting digestion pattern indicated successful donor-mediated HDR (Figure 1). Three candidate clones were identified (1 from ssODN, 2 from ssODN-Ph) using EcoRI screening, from 36 colonies screened (12.5% efficiency). The genotype of one clone (4A) with an EcoRI digestion pattern indicative of biallelic targeting, was confirmed by Sanger sequencing and shown to have homozygous modified alleles and was designated *NRL*$^{-/-}$ (mutation nomenclature obtained using https://www.mutalyzer.nl/, and described as follows:

NG_011697.1(NRL_v001):c.221_222insAATTC) p.(Trp74*).

Off-target sites for the CRISPR/Cas9 sgRNA predicted by the software Benchling.com, based on Hsu et al[56] were investigated by amplifying genomic regions of ± 500 bp around the first 4 sites (see suppl. Table 3). PCR was performed using MyFi Polymerase (Bioline BIO-21117, according to manufacturer's instructions) and PCR amplicons purified using Monarch® PCR & DNA Cleanup Kit T1030S and Sanger sequenced (Source Bioscience);

files (.ab1) were aligned to the genomic sequences using Benchling software. Single-nucleotide polymorphism (SNP) microarray analysis performed using HumanCytoSNP-12 v2.1 BeadChip Kit from Illumina and Genomestudio 2.0 confirmed the edited line retained a normal digital karyotype.

Retinal differentiation

Both parental MShef10 ESC and *NRL*$^{-/-}$ cells were subjected to retinal differentiation using a previously published protocol[28]. Briefly, cells were cultured in pluripotency supportive media, and were allowed to reach complete confluency; then they were cultured for 48h in media lacking FGF and TGFb, to induce differentiation. On day three, Neural Inductive Media (NIM) was added to promote forebrain identity, and media was replaced thrice weekly. After three weeks, cells begin to form 3D rounded neuroepithelial structures termed Optic Vesicles (OVs), which protrude from the monolayer and grow over a patch of cells that form a retinal pigmented epithelium (RPE). Between weeks 4-7 OVs were manually excised from the plate and cultured in suspension in low attachment 96-well plates in Retinal Differentiation Medium, supplemented with Taurine and Retinoic Acid. OVs from independent passages were collected at several differentiation stages: week 7-8 (early differentiation) weeks 14-17 (middle differentiation), and 20-25 as late time points.

Immunohistochemistry

Three to six OVs per sample were fixed in PBS/4% PFA for 30 minutes at R/T, followed by three washes with PBS of 5 minutes each. They were then cryopreserved by immersion in a PBS/30% sucrose solution for 1h at 4°C, or until sunk. Samples were embedded in OCT compound, frozen and stored at -80°C. 12μm cryosections were sliced, OCT compound removed by warming the slides for 10 minutes at 37°C, and blocked for 1h using

PBS/0.1% Triton-X and 10% species-specific serum. Primary antibodies were diluted to the appropriate concentration in blocking buffer, and incubated O/N at 4°C. Secondary antibodies were diluted in blocking buffer, and incubated for 2h at R/T. Nuclei were stained with a 5μg/ml DAPI solution for 5 minutes at R/T, and slides were mounted with ImmunoFluoroMount (GeneTex GTX3028) and sealed with nail polish. Slides were imaged using a LSM710 Zeiss Confocal microscope, images processed using Fiji software and figures assembled using Inkscape. Antibodies used are listed in Table 1.

VSX2 and Ki67-positive cell quantification

The number of VSX2 positive and Ki67 positive cells was quantified in three fields of view per OV. Cells were manually labelled using an ROI tool on the Icy bioimage software (http://icy.bioimageanalysis.org) on three channels (DAPI, blue; VSX2, Green; Ki67, red). ROIs were automatically optimised using the Active Contours plugin; green or red channel ROIs were superimposed onto the blue (DAPI) channel to exclude false positive signals; (N = 1 differentiation, n = 3 OVs; for each genotype). Co-localised ROIs for DAPI /VSX2 or Ki67/VSX2 were counted and the percentage VSX2+/Ki67+ cells were calculated.

S-OPSIN-positive cells quantification

The number of cells positive for S-OPSIN was quantified by randomly selecting three fields per OV structure using DAPI staining only to avoid bias, and then quantifying the number of positive cells stained with the specific antibody in a total of three fields per OVs, in nine or three samples of OVs from three or one independent differentiation experiments for *NRL*$^{-/-}$ and WT, respectively.

Quantitative Real-Time PCR

Organoids were analyzed from four independent retinal organoid differentiations (N) of *NRL*$^{-/-}$ ESCs and three control MShef10 ESC (WT) differentiations; one to three samples (n) per time point for each differentiation, each sample consisting of a pool of three OVs, were flash frozen in dry ice for RNA preservation. Three independent passages of undifferentiated ESCs (control MShef10 and *NRL*$^{-/-}$) were used as a day 0 control. RNA was extracted using the RNeasy Micro Kit (Qiagen, # 74004) adding the DNase treatment, and RNA measured using Nanodrop. 500ng of RNA was used for cDNA synthesis using the RevertAid H Minus First Strand cDNA Synthesis Kit (Thermo Scientific, # K1631). Quantitative Real-Time PCR (qRT-PCR) was performed in 20μl reactions, using 1μl cDNA, 0.25μM primers and SYBR™ Green PCR Master Mix (Thermo Scientific, #4309155). The reaction was run on a StepOnePlus™ Real-Time PCR System (Applied Biosystems, #4376600), with the parameters: 10' 95°C; 40x 15'' 95°C and 1' 60°C followed by melting curve analysis. GAPDH was used as reference gene and Ct values were analyzed as previously described using Graphpad Prism software. Primers sequences are in Table 2.

Statistical analysis

qRT-PCR and IHC data was analyzed using two-way ANOVA with multiple comparisons and Sidak's correction in Prism8 **: $p < 0.01$, ***: $p < 0.001$, ****: $p < 0.0001$. N = independent differentiation experiments; n = separate organoid samples; Sets of OVs from at least 2 independent differentiation experiments for each genotype were analysed by qRT PCR and replicate numbers (N, n) are indicated in the Figure legends.

Table 1. Antibodies list

Antibody	Cat. number	Concentration
**Primary**		
NRL	R&D AF2945	1:100
NR2E3	R&D H7223	1:500
Cone arrestin (ARR3)	Novus Biologicals NBPI-37003	1:100
CRX	Abnova H000014-06-M02	1:800
Recoverin	Abcam AB5585	1:1000
VSX2	Abcam AB9016	1:400
Ki67	Abcam AB15580	1:300
ZO-1	Thermofisher 61-7300	1:300
S-OPSIN	Abcam AB5407	1:350
L/M-OPSIN	Abcam AB5405	1:200
RXRγ	Abcam AB15518	1:200
Rhodopsin	Merk O4886	1:1000
Synaptophysin	Abcam AB8049	1:500
PSD95	Abcam AB18258	1:200
CACNA1F	Merk HPA068379	1:200
GFAP	Merk AB5804	1:500
RIBEYE (CtBP2)	BD 612044	1:500
PKCa	Merk P5704	1:10
SOX9	R&D AF3075	1:500

PAX6	Biolegend 901301	1:400
**Secondary**		
Alexa 594 Rabbit anti-goat	Thermofisher A11080	1:800
Alexa 594 Goat anti-mouse	Thermofisher A11032	1:800
Alexa 594 Donkey anti-rabbit	Thermofisher A121207	1:800
Alexa 594 Goat anti-rabbit	Thermofisher A11037	1:800
Alexa 488 Donkey anti-sheep	Thermofisher A11015	1:800
Alexa 488 Donkey anti-rabbit	Thermofisher A21206	1:800
Alexa 488 Donkey anti-mouse	Thermofisher A21202	1:800
Alexa 488 Goat anti-rabbit	Thermofisher A11008	1:800

Table 2. qRT-PCR primers list

Name	Sequence Forward	Sequence Reverse
GAPDH	AATCCCATCACCATCTTCCA	TGGACTCCACGACGTACTCA
CRX	GCCCCACTATTCTGTCAACG	GTCTGGGTACTGGGTCTTGG
VSX2	TCATGGCGGAGTATGGGCT	TCCAGCGACTTTTTGTGCATC
NRL	CATGGCTACTACCCAGGGAGC	GTTTAGCTCCCGCACAGACATC
NR2E3	AGCAGCGGGAAGCACTATG	CCTGGCACCTGTAGATGAGC
THRB	TGATGATGTGAACGACCAGAGT	AGTAACTGGGGATGTACCCTTT
RXRγ	CCGGATCTCTGGTTAAACACATC	GTCCTTCCTTATCGTCCTCTTGA
OPN1SW	CCTGGCTACCTGGACCATTG	TAGGACTCGCTGCGGTATTTG
OPN1MW	CATCCGCAGGACAGCTATGAG	GGAGCGATGTGGTAATTCGGG
ARR3	CGGTGGAACCCATTGACGG	TCCAAGTCATCACGGCCATAG
RHO	ACAGGATGCAATTTGGAGGGC	GCTCATGGGCTTACACACCA

MEF2C	CTGGTGTAACACATCGACCTC	GATTGCCATACCCGTTCCCT
GNGT1	TCCCAAGGCTAGTGTGCATTGCT	ACCAGTGGATCCTCGCCAGATCG
GRK7	TGCTGGGGAAAGGTGGTTTT	GTCCAGTTTCTTACAGGCATACA
PDE6H	GCAGACTCGCCAATTCAAGAG	TCTGTTCCTAGCCCCTCCATT

Table 3. CRISPR/Cas9 Off-target effects

Sequence	PAM	Score	Gene	Chr	Strand	Position	Mismatches	On-target
CAGGCCTGGAGGAGCTGTAC	TGG	100	ENSG00000129535	chr14	-1	24082633	0	FALSE
CAGG**T**CTGGAGGAGCTGTA**G**	AAG	5.659286		chr9	-1	132597790	2	FALSE
CAGGCCTG**T**AGGAGCTGT**C**C	AGG	1.662198	ENSG00000171298	chr17	1	80104569	2	FALSE
CAGG**G**CTGG**G**GGAGCTGTA**G**	AAG	1.474158		chr7	-1	43197557	3	FALSE
**G**A**AC**CCT**A**GAGGAGCTGTAC	CAG	1.457521		chr1	-1	6462730	4	FALSE

Table 4. Primers to amplify off-target regions

Off-target site	Sequence Forward	Sequence Reverse	Amplicon (bp)
# 1	TAATTGCCTACAGAAGCGGTGC	AGCCAGGAAAGGTGGTGATGAT	359
#2	CCTGCTGGAGCTTTTCTCGCCC	TGCTGGTGAGCTGGGTGAGTCT	403
#3	TCCGCTGCCCTAGACCACCTTC	GGGCCACGGTTATGTTGCCAGT	395
#4	CGTGCCAAGCTCCAGGGATCAT	CCTTGTGGTCCCCCTCCTGCTT	396
